# Electrodeposition of Samarium Metal, Alloys, and Oxides: Advances in Aqueous and Non-Aqueous Electrolyte Systems

**DOI:** 10.3390/ijms262211176

**Published:** 2025-11-19

**Authors:** Ewa Rudnik

**Affiliations:** Faculty of Non-Ferrous Metals, AGH University of Krakow, al. Mickiewicza 30, 30-059 Krakow, Poland; erudnik@agh.edu.pl

**Keywords:** samarium, deep eutectic solvents, ionic liquids, molecular liquids, molten salts, electrodeposition, electrowinning, recovery, hydrometallurgy, solvometallurgy

## Abstract

Samarium, a rare earth element, is crucial for advanced technological applications, particularly due to the exceptional magnetic properties of Sm_x_Co_y_ intermetallics, discovered over 50 years ago. However, its growing significance and demand have highlighted concerns about scarce, commercially viable natural sources and the complex separation processes needed to isolate it from other lanthanides. In this context, electrodeposition has emerged as a versatile method for both synthesizing samarium materials and recovering the element. A major obstacle in applying electrolysis lies in the complex electrochemical behavior of samarium species, stemming from their highly negative electrochemical potential. While this limits the use of aqueous solutions, it also opens up possibilities for alternative solvents, such as molecular liquids, ionic liquids, deep eutectic solvents, and molten salts. The electrochemical properties of samarium have prompted exploration into electrodeposition techniques for material synthesis and recycling. This review discusses various aqueous and non-aqueous electrolyte compositions, different electrolysis modes, and the role of cathode substrates. It also shows the potential of electrolysis in the fabrication of various cathode products (metal, alloys/intermetallics, inorganic compounds), highlighting both challenges and opportunities in its practical implementation.

## 1. Introduction

Samarium, as a rare earth element, is considered a critical and strategic mineral by various economies worldwide, including Australia, Brazil, Canada, China, the European Union, India, the United Kingdom, and the USA [1]. Its commercial importance emerged in the mid-1960s, when Karl J. Strnat and coworkers (USA) identified the exceptionally high magnetocrystalline anisotropy of rare earth–cobalt alloys [2]. This discovery led to the development of samarium–cobalt intermetallic compound SmCo_5_ of the high-energy product (119–160 kJ/m^3^) [3,4]. It represented a major milestone [5] in the progress of magnetic materials and paved the way for the fabrication of other high-performance samarium-based permanent magnets [6], such as the Sm_2_Co_17_ type (159–264 kJ/m^3^) in the 1970s (USA) [4,7] or samarium–iron–nitride Sm_2_Fe_17_N_3_ (160 kJ/m^3^) in the 1990s (Japan) [8,9]. Although samarium-cobalt permanent magnets represent niche market materials (accounting for 0.4% by weight and 2.1% by value in 2020 [10]), their market value is currently estimated at approximately 500 million USD (2025), with robust growth projected to reach about 850 million USD by 2033 [11]. A significant increase in the market share of these magnets is not expected (0.3% in 2030, i.e., 4600 t [10]), primarily due to limited samarium availability [12], high production cost compared to other permanent magnets (NdFeB, ferrite, Alnico) [10,13], as well as brittleness, limited mechanical strength, and the low corrosion resistance of iron-containing magnet variants [9]. However, unique advantages of samarium-based magnets are their high-temperature stability, which enables operation within the temperature range of 250–500 °C, owing to an exceptional resistance to demagnetization and a Curie temperature of 700–800 °C [4,6,14]. This opens possibilities for their use in specialized applications that include the aerospace and defense industries (e.g., high-precision applications such as aircraft actuators and guidance systems), the automotive sector (high-performance electric motors and generators in electric and hybrid vehicles), medical devices (e.g., magnetic resonance imaging machines, precision instruments), green energy devices (e.g., turbo machines, wind turbines), advanced electronic devices (i.e., robots, automation equipment, sensors) [4,9,11].

**Figure 1:**
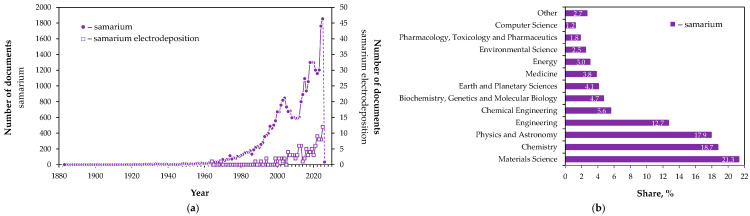
Scopus-indexed publications (1883–2026): (**a**) number documents with the keywords “samarium” and “samarium electrodeposition”, (**b**) distribution of documents by subject area with the keyword “samarium”. Data source: Scopus, Elsevier [26] (retrieved on 17 October 2025).

While samarium-based magnets accounts for the vast majority of its industrial use (97% in the EU [15]), the remaining share involves other fields, such as medicine (^153^Sm isotope for bone cancer therapy [16]) and optics (as an oxide-dopant in optical fibers, lasers, display units, and infrared detectors [17,18]). Moreover, samarium serves as a neutron absorber (^149^Sm and ^152^Sm isotopes) in control rods of nuclear reactors [18,19], contributes to energy-efficient lighting [17], its compounds act as catalysts in organic (electro)synthesis [20,21,22], and plays a role in geological dating (^147^Sm isotope) [23,24]. This significant progress in samarium research and industrial implementation has been observed since the late 20th century, about 100 years after the element was first discovered (1879) and isolated as pure oxide (1901) and in metallic form (1903) [24,25]. Such interest is evidenced by the marked increase in scientific publications indexed in the Scopus database [26] under the keyword “samarium” (Figure 1).

The development of samarium materials science is closely tied to advances in methods (physical, chemical, electrochemical) for producing the metal, its alloys, and chemical compounds. Among these, electrodeposition stands out as a versatile technique for fabricating layers, thin films, and diverse (nano)structures, which are essential for the manufacture of innovative functional materials, the miniaturization of devices, and the precise engineering [27,28]. This process relies on the cathodic reduction of ions from aqueous or non-aqueous electrolytes (molecular liquids, ionic liquids, deep eutectic solvents, molten salts) and allows accurate control over material composition, thickness, surface morphology, and properties often unattainable by other methods. Beyond the fabrication of engineering materials, electrodeposition is also employed in electrowinning or electrorefining, the final stage of metallurgical production, where metal is separated and recovered from primary or secondary sources [27].

Although not the major method for producing samarium [29,30,31] and samarium-containing materials [29,31], the electrodeposition has attracted growing scientific attention over the past two decades (Figure 1a). Its advantages and versatility have inspired the present review, which aims to examine its potential practical applications, while also addressing the challenges and limitations of its implementation. In the context of increasing interest in rare earth metals, exploring the capabilities of electrochemical methods as alternative approaches for samarium recovery (from natural and waste sources, including nuclear materials) or for the fabrication of various samarium-containing materials (magnetic, semiconducting, catalytic, protective, radioisotope-target) using different electrolyte systems is essential. Such insight not only broadens the scientific perspective on the element’s applications but also underpins the progress in advanced functional materials and sustainable metal recycling technologies.

## 2. Electrodeposition of Samarium Metal

### 2.1. Aqueous Solutions

**Figure 2:**
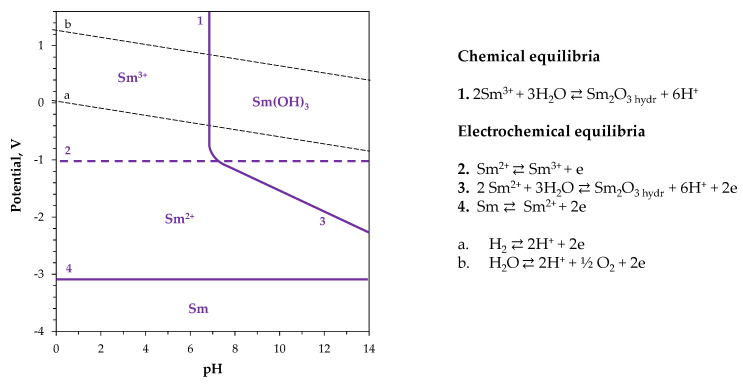
E-pH diagram for Sm-H_2_O system. Adapted from [32].

In aqueous solutions of simple salts (e.g., chloride, nitrate, sulfate), samarium predominantly exists in the trivalent form as Sm^3+^ ions [32], which are thermodynamically stable under normal conditions (Figure 2). Divalent Sm^2+^ ions can be generated through the reduction of Sm^3+^ species and cyclic voltammetric studies proved this as reversible electrode reaction [33,34]. Atanasyants and Seryogin [35] reported that the efficiency of electrochemical converting trivalent to divalent samarium ions in hydrochloric acid solutions can reach up to 85%, and is influenced by solution acidity, current density, temperature, and agitation rate. It is worth noting that this partial reduction process can serve as a potential intermediate step in the extraction of samarium from an aqueous mixture of rare earth elements, yielding the final crystallized sulfate salt, SmSO_4_.

The Sm^2+^ ions are unstable in water and oxidize back to trivalent state due to their strong tendency to react with dissolved oxygen or protons [36]. This behavior corresponds to the very low electrochemical potential of the Sm^3+^|Sm^2+^ redox couple of −1.33 V vs. SHE (Table 1). Furthermore, the extremely negative reduction potentials of the Sm^3+^|Sm^0^ and Sm^2+^|Sm^0^ pairs (below −2 V vs. SHE) make the direct electrodeposition of metallic samarium from aqueous electrolytes practically impossible, as hydrogen evolution occurs preferentially under such conditions. Consequently, the electrolyte becomes highly alkaline at the cathode surface [37], leading to the secondary precipitation of hydrated samarium oxide (or samarium hydroxide). This process is described in more detail in Chapter 4.

Nevertheless, Lokhande et al. [39,40] reported the electrodeposition of samarium metal from aqueous baths containing complexing agents on various substrates, including copper, brass, titanium, stainless steel, and ITO-coated glass. They demonstrated that the addition of oxalate, citrate, and thiocyanate ligands to samarium nitrate solutions shifted the deposition potential toward more positive values, whereas EDTA showed the opposite effect [39]. Oxalic acid was identified as the most effective bath additive, exhibiting the strongest influence on the deposition potential. Further studies [40] investigated the effects of pH (2–12), tartaric acid concentration (0.1–1M), temperature (25–60 °C), deposition time (5–30 min), and substrate type on samarium deposition from tartrate baths. Thin (up to 4 µm), white-gray, and uniform deposits were obtained from acidic solution (pH = 2) with high acid concentration (0.05M Sm_2_O_3_, 1M C_4_H_6_O_6_) and at low current densities (up to 0.5 A/dm^2^). Interestingly, the deposition rate typically decreased with electrolysis time (for first 10 min) for all substrate types, significantly affecting the final coating thickness. Although the influence of deposition parameters on cathodic reaction kinetics and film morphology was examined, the formation of a metallic samarium layer was not unambiguously confirmed. Thus, it remains questionable whether the metal deposit, as stated by the authors [37,38], was indeed obtained.

### 2.2. Molar Liquid Electrolytes

**Figure 3:**
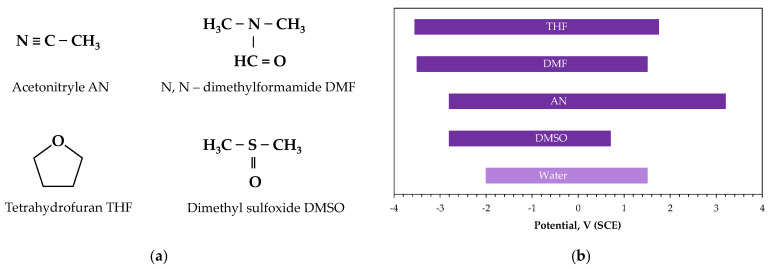
Molar liquid solvents: (**a**) structural formulas, (**b**) electrochemical windows (adapted from [41,43]).

Molar liquid solvents represent conventional non-aqueous media commonly employed for the electrodeposition of metals and alloys [41,42]. These include polar organic compounds (Figure 3a), both aprotic (such as dimethylformamide DMF, dimethyl sulfoxide DMSO, acetonitrile AN, tetrahydrofuran THF, acetone) and protic (absolute alcohols, e.g., ethanol, methanol). Such solvents are relatively inexpensive, readily available on an industrial scale, and exhibit advantage physicochemical properties like low viscosity, high metal salt solubility, good thermal and electrochemical stability. They exhibit a wide electrochemical window (Figure 3b), which is particularly important, as it mitigates the issue of preferential hydrogen evolution during the electrodeposition of active metals, including rare earth elements [40].

Pioneering studies on samarium electrodeposition in molecular liquid solvent were recently reported by Gao et al. [44] as a potential approach for solvometallurgical metal electrowinning. They found that electroreduction of samarium to its metallic form did not occur (Al or W substrate, 25 °C) in the anhydrous SmCl_3_–DMF system (0.05M Sm^3+^), even after 90 min of potentiostatic electrolysis at a voltage gradient from −1.5 V to −3.5 V vs. Ag. This inability to deposit metallic samarium was attributed to the formation of highly stable [Sm(DMF)_n_]^3+^ complexes:

SmCl_3_ + nDMF → [Sm(DMF)_n_]^3+^ + 3Cl^−^ (1)

The addition of lithium nitrate LiNO_3_ as a supporting electrolyte significantly enhanced the electrodeposition of samarium by increasing the overall electrolytic conductivity and reducing the solvation number of samarium ions. Moreover, the nitrate ions NO_3_^−^ weaken the strong coordination of DMF molecules with Sm^3+^ ions, leading to the formation of electroactive complexes [Sm(DMF)_x_Li(DMF)_y_NO_3_(DMF)_z_]^3+^ that can be more easily reduced at the cathode. Cyclic voltammetry studies revealed that overall reduction of samarium species takes place in two distinct stages: initially from the trivalent to the divalent state, and subsequently to the elemental form:

[Sm(DMF)_n_]^3+^ + e → [Sm(DMF)_n−1_]^2+^ + nDMF (2)

[Sm(DMF)_n−1_]^2+^ + 2e → Sm + (n − 1)DMF (3)

This two-step mechanism differs from the typical behavior of most lanthanides, which are usually reduced in a single three-electron transfer process.

Samarium was electrodeposited (Al substrate, −2.8 V vs. Ag) as an adhesive black-gray coating, exhibiting a dense and uniform granular structure, although some cracks were observed at high magnifications. XPS analysis unambiguously confirmed the presence of metallic samarium, along with its oxide, which could have formed as a secondary contaminant due to samarium’s tendency to oxidize upon exposure to air. Although these investigations were primarily intended for electrolytic samarium recovery, key indicators such as current efficiency and energy consumption were not estimated.

### 2.3. Ionic Liquid Electrolytes

Ionic liquids have emerged over the past two decades as an innovative class of solvents used in electrodeposition processes [42,45]. They are considered “green systems” [46], offering an alternative to traditional electrolytes such as aqueous solutions, molecular organic solvents, or high-temperature molten salts [47]. Ionic liquids typically consist of large organic cations combined with smaller organic or inorganic anions [48]. These compounds exhibit a wide liquid range with melting points below 100 °C, high solubility for metal salts, good chemical and thermal stability, as well as moderate-to-high electrical conductivity (up to 0.1 S/m) and broad electrochemical windows (2–8 V) [47,48]. The main drawback of these solvents, however, lies in their complex synthesis [48] and purification methods [47,49], which make ionic liquids relatively expensive.

**Figure 4:**
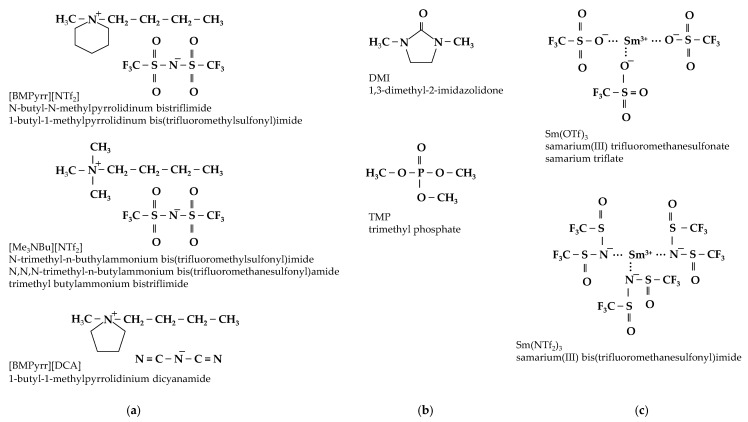
Components of ionic liquid-based electrolytes for samarium electrodeposition: (**a**) ‘conventional’ ionic liquids, (**b**) neutral ligands, (**c**) samarium(III) salts.

**Figure 5:**
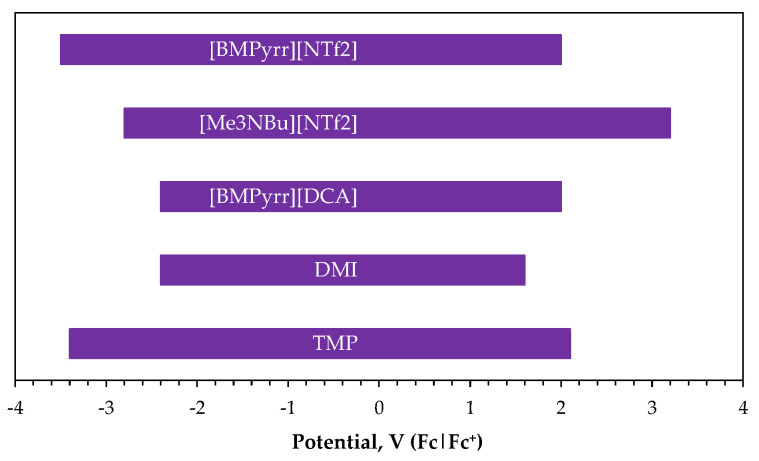
Electrochemical windows of ionic liquids used for samarium electrodeposition. Adapted from [58,67,68].

Ionic liquids have also gained importance in the electrodeposition of rare earth metals [50,51]. Although the number of studies on Sm^3+^ electroreduction from ionic liquids is limited [52,53,54,55,56,57,58,59,60,61,62,63,64,65,66], several have focused primarily on investigating the Sm^3+^|Sm^2+^ redox couple in the context of spent nuclear fuel treatment or redox battery applications [55,61,62,63,64]. Nevertheless, the available research demonstrates the feasibility of metallic samarium formation [52,56,57,58,59,60,62] from both ‘conventional’ ionic liquids (Figure 4a) and a sub-class known as neutral-ligand ionic liquids (Figure 4b). All of these systems exhibit high stability in wide ranges of potentials (Figure 5).

The electrolytes can be prepared either by dissolving commercially available samarium compounds [52,53,58,60,61,65,66] or by using self-synthesized salts obtained from Sm_2_O_3_ [54,55,56,57,59,63], which were subsequently dissolved in the appropriate ionic liquids. Another approach involves the anodic dissolution of metallic samarium directly in the ionic liquid [62]. Samarium compounds, such as Sm(OTf)_3_ or Sm(NTf_2_)_3_ (Figure 4c), are commonly used due to their high solubility, although simple salts like nitrate or chloride have also been investigated. Since the deposited metal is highly sensitive to moisture, the water content in the electrolyte must be kept at a trace level, determined experimentally (e.g., 0.9 ppm [56], 169 ± 10 ppm [53]) or controlled using a glove box system, typically maintaining levels below 10 ppm [57,58,59,60] or 50 ppm [52,63]. Molodkina et al. [66] showed that the addition of controlled amounts of water (0–3M) shifts the reduction potential of samarium ions in the [BMP][DCA] ionic liquid towards more electropositive potentials, while simultaneously promoting the co-deposition of hydroxide on the cathode.

Spectroscopic studies [57,58,59,60] revealed that samarium(III) exists in the ionic liquid electrolytes as complexes with organic ligands. For example, Andrew et al. [59] showed that in the Sm(NTf_2_)_3_–[BMP][NTf_2_] system, samarium forms a [Sm(NTf_2_)_4_]^−^ complex. In [BMP][DCA] solutions, samarium ions consistently form complexes with DCA^−^ anion through the nitrogen atom of dicyanamide group [57]. This behavior was observed independently of the metal salt used (triflate, nitrate, or chloride), and the presence of [Sm(DCA)_x_(L)_y_]^3−^ (where L is OTf^−^, NO_3_^−^, or Cl^−^) was postulated as the predominant species. In turn, in neutral ligand-based ionic liquids, samarium ions are coordinated by neutral organic ligands NL and paired with ionic liquid anions ILA to form neutral ion pairs [Sm(NL)_x_]^3+^[(ILA)_y_]^3−^. These ion pairs serve a dual function: the neutral ligand enhances the solubility of metal ions, while the ionic liquid medium facilitates their reduction to the metallic state [67]. Andrew et al. [57] identified [Sm(TMP)_3_]^3+^ cations in the Sm(NTf_2_)_3_–TMP system, where Sm^3+^ is coordinated through the oxygen atom in the P=O group. Conversely, the stoichiometry of the complex in the DMI solvent [60] was dependent on the samarium(III) salt used, with the existence of [Sm(DMI)_3_]^3+^ and [Sm(DMI)_4_]^3+^ being proposed for Sm(NO_3_)_3_·6H_2_O and Sm(OTf)_3_ precursors, respectively.

Reduction of samarium species in ionic liquids has been confirmed to occur in two consecutive stages: the first involves a quasi-reversible one-electron reaction of Sm^3+^ to Sm^2+^ [57,61,62,63], followed by an irreversible two-electron process of Sm^2+^ to Sm^0^ [54,55,57,58,59,60]. However, cyclic voltammetric CV experiments cannot always clearly identify the second stage (Table 2) due to the potential overlap with the electrochemical solvent degradation [53,66]. On the other hand, some studies [52,65] reported cathodic signals in [BMP][NTf_2_] electrolytes, but no anodic response, interpreting this as irreversibility of the reduction of Sm^3+^ species to Sm^0^. In contrast, Pan et al. [62] observed that in 1-butyl-3-methylpyrrolidinium bis(trifluoromethylsulfonyl)imide [Me_3_BuPyrr][NTf_2_], reduction of Sm^3+^ to Sm^2+^ occurs within a short time of CV tests, but over extended periods, Sm^2+^ undergoes disproportionation:

3Sm^2+^ → 2Sm^3+^ + Sm (4)

However, any metallic samarium produced during this process reacted with the ionic liquid, showing instability of metallic form in this medium.

Such samarium behavior was further verified by Manjum et al. [56] in the Sm(NTf_2_)_3_–[BMP][NTf_2_] ionic liquid at elevated temperature (100 °C). Although electrochemical deposition of samarium was not observed on a glassy carbon electrode (at −1.5 V and −2.5 V vs. Ag/Ag^+^), the reaction (4) led to the formation of metal nanoparticles dispersed throughout the bulk electrolyte, resulting in the deposition of a granular film composed of these nanoparticles. At a lower temperature (25 °C), no deposit was formed on the electrode surface.

Table 2 summarizes the conditions for samarium electrodeposition, including various electrolyte compositions, deposition parameters, and substrates. Typically, potentiostatic deposition is employed for better control of the process by adjusting the electrode potential within the electrochemical window of the ionic liquid. Samarium forms deposits up to a few micrometers thick, featuring a generally rough surface, consistently exhibiting network of cracks [52,57,58,59,60,65]. While the metallic phase was unequivocally confirmed in the coatings, some amounts of oxygen was also identified. This was attributed to secondary oxidation of the deposit’s surface upon exposure to air.

An influence of the ionic liquid type on the morphology of the cathodic deposits is evident, although the differences may stem from varying electrolysis conditions. Andrew et al. [58,60] conducted comparative studies on the effect of different samarium salts in ionic liquids. Samarium deposits obtained from the Sm(OTf)_3_–DMI electrolyte exhibited a more compact structure, while non-uniform granular deposits were formed from the Sm(NO_3_)_3_·6H_2_O–DMI system [60]. The addition of Sm(OTf)_3_, Sm(NO_3_)_3_·H_2_O, or SmCl_3_ into [BMP][DCA] further emphasized the role of salt anions in samarium deposition across varying temperatures (25–60 °C) and cathode potentials [58]. Smoother deposit surfaces were achieved at lower temperatures and less cathodic potentials, although the presence of hydrated nitrate salt negatively affected the compactness of the coating. Ispas et al. [65] observed that galvanostatically deposited films were smoother compared to those deposited potentiostatically. Furthermore, thin layers deposited under potentiostatic conditions exhibited more cracking than thicker ones.

Unfortunately, despite the fact that many of these deposition processes were inspired by potential applications in metal electrowinning, no current efficiency values were reported in any of the studies, which would have allowed for an assessment of the potential for practical implementing this method.

### 2.4. Deep Eutectic Solvent Electrolytes

Deep eutectic solvents were first employed in the early 2000s and quickly gained widespread use as a medium for the electrodeposition of metals and alloys [69]. These liquids are eutectic mixtures of two or more molecular compounds, which act as hydrogen-bond acceptors combined with hydrogen-bond donors [70,71]. They have a melting point lower than that of their individual components and also lower than that of an ideal liquid mixture [70]. Deep eutectic solvents share many advantageous physicochemical properties with ionic liquids (e.g., wide liquid range, low volatility, high solubility for both inorganic and organic compounds), and their simple preparation process (by mixing components with moderate heating) makes them significantly easier and more cost-effective to produce [72]. However, it is important to note that they are more viscous than conventional aqueous and non-aqueous solvents, are not resistant to elevated temperatures and may start to decompose at about 100 °C [71,73]. Additionally, they are sensitive to water and air, requiring a controlled atmosphere during use [69,70].

**Figure 6:**
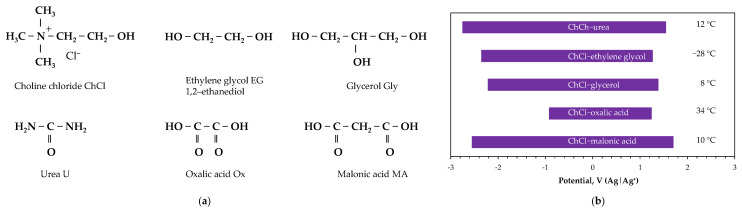
Deep eutectic solvents: (**a**) structural formulas of components, (**b**) electrochemical windows on GC electrode (adapted from [74]) and melting points of eutectics (adapted from [70,75]).

Most deep eutectic solvents used for metal electroplating purposes are formed by combining choline chloride with urea, ethanediol, or glycerol [69,71,73], which exhibit an electrochemical window wider than that of aqueous systems (Figure 6). However, it should be emphasized [73] that these systems exhibit relatively low deposition rates, typically one order of magnitude lower than in conventional aqueous baths. Thus the operating temperature should be maintained at a slightly higher level to ensure low viscosity and high conductivity of the bath. They also have relatively poor ability to achieve uniform deposition thickness over the substrate (throwing power) and lose their properties at sufficiently high water contents, which is especially important when depositing reactive metals. Additionally, there are challenges in electrolysis with insoluble anodes due to the breakdown of solvent components. All of these factors make deep eutectic solvents difficult to adopt in practical electrodeposition applications.

Electrodeposition of rare earth metals from deep eutectic solvents has recently gained increased attention, although the process of depositing pure metals has not advanced significantly, primarily due to the decomposition of organic bath components before metal deposition [69,76]. As a result, only a few reports have been found related to samarium electroreduction [77,78,79,80]. The only successful samarium deposition (Table 3) was conducted by Gómez et al. [77], using a mixture of metal nitrate in a choline chloride–urea solvent. Cyclic voltammetry, galvanostatic and potentiostatic experiments confirmed the formation of a metal deposit. They found that the potential at which solvent reduction occurred was significantly more negative than the potential at which the Sm^3+^ ion was reduced, indicating metal formation as the main process. Samarium deposits produced after a voltammetric hold at potential value (−1.45 V vs. Ag/AgCl) corresponding to the first voltammetric peak were fine-grained at short deposition time (15 min), while longer times (25 or 80 min) led to increased coverage, cracks, new growth on the initial deposit and caused further cracking. Deposits obtained in potentiostatic conditions exhibited similar morphology, but when a more negative potential (−1.9 V vs. Ag/AgCl) was applied, smooth deposits were formed.

Other deep eutectic solvent systems, such as choline chloride–ethylene glycol [78] and urea–acetamide–alkali bromides [79,80], were also examined, but no reduction of samarium species to their metallic form was observed through cyclic voltammetry [78,79,80] or potentiostatic deposition [79]. In all these cases, the electrochemical windows determined in metal-free baths were around 2 V, which met the requirements for the possible reduction of metal ions. Notably, the unsuccessful experiments were conducted on platinum substrates [78,79,80], while the only successful metal deposition was achieved on a glassy carbon cathode [77]. This can emphasize the role of the substrate material in the kinetics of electroreduction reactions, which are influenced by the cathodic overpotential of the electroactive species in the electrolyte (e.g., high hydrogen overpotential on glassy carbon, but low on platinum).

### 2.5. Molten Salt Electrolytes

Molten salt-based electrodeposition is a common method for producing active metals that cannot be electrowon from aqueous solutions [27]. This technique has been implemented industrially as the final stage of rare-earth element production for over a century, owing to its high efficiency and convenience compared with thermal processes, despite the high operating temperatures required (700–900 °C for chlorides and 1100–1700 °C for fluorides) [29,30]. However, high-temperature electrolysis present significant challenges in the recovery of metallic samarium due to the electrochemical properties of samarium species in these systems strongly influenced by the material of the cathode substrate and melt composition. As a result, metallothermic reduction remains the primary industrial method for large-scale production of this metal [81]. On the other hand, molten salt electrolysis appears to be a promising pyrochemical method for the selective separation of samarium from other lanthanides in spent nuclear fuel (lanthanides are products of uranium fission) [82,83,84,85,86,87,88,89,90,91,92,93,94,95,96,97,98,99] of next-generation reactors (Gen IV) [100] or formation of samarium alloys in simple Sm^3+^–alkali salts systems [101,102,103,104,105].

Depending on the type of alkali metal salt used as the molten electrolyte, samarium(III) ions exist as chloride or fluoride complexes of varying stoichiometry and stability [96,98]. For example, SmCl_4_^−^, SmCl_6_^3−^, Sm_2_Cl_8_^2−^, and Sm_2_Cl_7_^−^ species were identified in the SmCl_3_–LiCl–KCl system, and their concentrations vary with time (at constant temperature) [96] and with temperature (at constant SmCl_3_ content) [96,98]. This behavior was attributed to conversion processes involving the formation of complexes with different coordination numbers:

5SmCl_6_^3−^ + 3Sm^3+^ ⇄ 2SmCl_4_^−^ + Sm_2_Cl_8_^2−^ + 2Sm_2_Cl_7_^−^ (5)

Samarium ions also form six-coordinated species in fluoride-containing melts. The stronger coordination of cation with fluoride than chloride ligands allows F^−^ to partially replace Cl^−^ in mixed salt systems [98]. Thus, a more stable fluoride coordination sphere leads to slower charge transfer during reduction reactions in chloride–fluoride or fluoride melts.

Interestingly, unlike in aqueous solutions, samarium forms both stable and soluble divalent and trivalent ions in molten systems. This distinguishes it from most other lanthanides, which exist in such media exclusively in the trivalent state. Bae et al. [97] confirmed the higher stability of the divalent oxidation state compared with the trivalent one in the LiCl–KCl system, noting that the Sm^2+^ (as chloride complex) may persist even after cooling of the melt. Castrillejo et al. [92] reported that dissolution of Sm_2_O_3_ in chloride melts (eutectic LiCl–KCl and equimolar NaCl–CaCl_2_) leads to the formation of stable SmOCl, which can efficiently converted into soluble form by chlorination with gaseous HCl in the molten medium.

Samarium ions are reduced from molten electrolytes (Table 4) either in a single step (to Sm^2+^) or in two consecutive steps (to Sm^0^). Divalent ions are reduced to form alloys on reactive electrodes, including both solid substrates (e.g., Ni [82,88,95], Cu [84,88,95], Al [83,84,88,95], Co [103,104], Fe [105]) and liquid cathodes (e.g., Pb–Bi eutectic [85], Sn–In alloy [93], Ga [86], Zn [95]).

The reduction of Sm^2+^ species proceeds through underpotential deposition, resulting from the depolarizing effect associated with the formation of M_x_Sm_y_ intermetallic compounds:

*x*Sm^2+^ + 2*x*e + *y*M → Sm_x_M_y_ (6)

where M is metal of reactive cathode substrate.

The intermetallic formation occurs regardless of the electrolysis mode (potentiostatic or galvanostatic) [95,101], with the resulting depolarization effect dependent on the cathode material in the following order: Cu < Ni < Al < Zn_liq_ in LiCl–KCl melt. This observation is particularly important for improving current efficiency in molten salt systems, although such data have not been reported in the literature. Ida et al. [101] demonstrated that the formation rate of the Ni_2_Sm intermetallic compound on a nickel electrode in the SmCl_3_–LiCl–KCl system was significantly higher under galvanostatic than potentiostatic conditions. The accelerated alloy growth was attributed to the concurrent deposition of lithium metal, since a slightly lower potential was reached during current-controlled electrolysis (−0.03 V compared to 0.1 V vs. Li/Li^+^). Further studies [102] revealed that the alloy film grew linearly with time, with the rate initially limited by the supply of samarium species and later by diffusion through the solid product layer. Similar results were obtained for the Co_2_Sm intermetallic compound on cobalt substrates (plate, nanoparticles), showing that the transient liquid lithium layer enhances the process by facilitating the formation of the L_x_Sm_4_Co_6_ (x ~ 3) product [103,104]. This ternary compound can decompose as follows:

Li_x_Sm_4_Co_6_ → Sm^2+^ + *x*Li^+^ + 3SmCo_2_ + (2+*x*)e (7)

Yan and Guo [105] synthesized the Sm_2_Fe_17_ alloy as a precursor for the prospective magnetic compound Sm_2_Fe_17_N_x_. The alloy was obtained using a molten mixture of calcium chloride and fluoride, enabling the incorporation of samarium into an iron substrate. The thickness of the alloy layer increased with deposition time, being controlled either by a Sm^3+^ diffusion-limited reaction at lower samarium chloride concentrations in the bath (below 0.017M) or by the diffusion of iron atoms through the intermetallic layer at higher samarium salt concentrations (above 0.017M). Moreover, the rate-determining step was found to be time-dependent at a given deposition potential, with the process governed initially by the cathodic reaction and subsequently by Sm^2+^ diffusion in the molten bath or iron atom diffusion in the solid state.

Nevertheless, pure metal is not deposited on the reactive electrodes, this electrolysis mode has proven effective for the selective separation of samarium from certain lanthanide ions, such as europium (copper substrate) [84].

In contrast, the electrodeposition of metal on inert solid cathodes (metals that do not form intermetallic compounds with samarium) is not feasible in both chloride and fluoride systems (Table 4), as its reduction potential exceeds the electrochemical stability window of the molten salt. Consequently, on inert substrates like Mo [82,83,86,90,91] or W [87,88,92], electrolyte decomposition occurs (reduction of alkali ion to metal) before samarium ions can be reduced to the metallic state. Castrillejo et al. [89] suggested that, at potentials similar or more negative than the Li/Li^+^ redox couple, metallic samarium in contact with a chloride melt can react with alkali metal ions according to the following reaction:

Sm + 2Li^+^ + 2e → Sm^2+^ + 2Li (8)

Notably, Manamura et al. [87] stated the possible formation of samarium metal on a gold inert electrode due to underpotential deposition, although this phenomenon was not investigated in detail.

While metallic samarium cannot be deposited on inert cathodes, electrolysis leads to the formation of soluble divalent samarium species from the trivalent ions. This reaction occurs at a more positive potential than the reduction of some lanthanides, allowing these elements to be selectively recovered in metallic form (intermetallic) from a melt containing a mixture of ions, while samarium remains in its ionic state in the molten bath. This strategy has been successfully demonstrated for the selective extraction of dysprosium (liquid Pb–Bi alloy substrate) [85], europium (aluminum substrate) [84] and erbium (liquid In–Sn alloy substrate) [93] in chloride systems.

## 3. Electrodeposition of Samarium Alloys

Obtaining pure samarium by direct electrolysis is not straightforward; however, it can be codeposited as component of an alloy. The presence of other metal ions in the electrolyte promotes the co-reduction of samarium species, with the mechanism being influenced by the speciation of samarium in various electrolyte types. In aqueous solutions, abnormal codeposition [106], specifically induced by ions of iron group metals (Co, Ni, Fe), is primarily observed. The process of alloy formation becomes more complex in non-aqueous organic baths. In turn, reaction (6) still occurs after the alloying element has been reduced to its metallic state in molten alkali salt electrolytes. These mechanisms of alloy codeposition are discussed in more detail in the following sections of this chapter.

A key factor is undoubtedly the formation of intermetallic samarium compounds, which significantly affect the deposition potential. During the simultaneous reduction of Sm^3+^ and M^n+^ ions, as generally described by the following equations:

Sm^3+^ + 3e → Sm (9)

M^n+^ + *n*e → M (10)

a solid alloy is produced:

*x*M + *y*Sm → M_x_Sm_y_ (11)

This leads to a shift in the quasi-equilibrium potentials of the metals, driven by the free energy Δ*G* of reaction (11) [107]:

(12) $$∆G=-RTln\frac{a_{M_{x}{Sm}_{y}}}{a_{M}^{x}\cdot a_{Sm}^{y}}$$

where *a_MxSmy_*, *a_M_*, and *a_Sm_* are activities of M_x_Sm_y_, alloying metal, and samarium in the deposit, respectively. For a single-phase M_x_Sm_y_ deposit, the activity *a_MxSmy_* may be considered equal to 1; therefore:

(13) $$a_{M}^{x}\cdot a_{Sm}^{y}=exp\left(\frac{∆G}{RT}\right)$$

The activities of both components in the deposit are interdependent, as an increase in the activity of samarium corresponds to a decrease in the activity of the other element. In the simplest case, with M_x_Sm_y_ as the only compound in the system coexisting with the pure metals (i.e., M/M_x_Sm_y_ and Sm/M_x_Sm_y_), relationships must be satisfied: $a_{M}=1$, $a_{Sm}=exp\left(\frac{∆G}{yRT}\right)$ and $a_{Sm}=1$, $a_{M}=exp\left(\frac{∆G}{xRT}\right)$. This induces a shift in the quasi-equilibrium potentials of the metal electrodes:

(14) $$E_{o,M}=E_{M}^{o}+\frac{RT}{nF}lna_{M^{n+}}-\frac{∆G}{nxF}$$

and

(15) $$E_{o,Sm}=E_{Sm}^{o}+\frac{RT}{3F}lna_{{Sm}^{3+}}-\frac{∆G}{3yF}$$

where $E_{M}^{o}$ and $E_{Sm}^{o}$ are the standard electrode potentials for alloying metal and samarium, respectively; $a_{M^{n+}}$ and $a_{{Sm}^{3+}}$ represent the activities of the corresponding metal ions at the electrolyte/deposit interface during electrodeposition.

Despite the increasing interest in electrodeposited samarium alloys from different electrolyte media, the number of studies still remains limited. However, these research are still driven on one hand by the unique magnetic properties of Sm–iron group metal alloys, and on the other by the selective separation of lanthanides from spent nuclear fuel.

### 3.1. Sm–Co Alloys

Sm–Co alloys in the form of films or nanostructures for magnetic material applications can be produced using various types of electrolytes [108,109,110,111,112,113,114,115,116,117,118,119,120,121,122,123,124,125,126,127,128,129,130,131,132,133], although in practice their composition shows rather limited diversity (Table 5).

#### 3.1.1. Aqueous Solutions

The typical bath formulation (ranging from acidic to neutral) used for Sm–Co alloy deposition contains sulfamate ions NH_2_SO_3_^−^ [108,109,110,111,112,113,114,115,116,117,118,119,120], although chloride [122] and chloride–sulfate baths have also been employed [123,124]. The significant difference in the deposition potentials of cobalt and samarium makes their simultaneous electrodeposition challenging, despite the expected potential shift resulting from the formation of intermetallic compounds. Consequently, metallic cobalt or its hydroxide/oxide together with samarium hydroxide/oxide tends to form on the cathode surface [110,119] that requires further metallothermic reduction [119]. To mitigate this problem, the addition of complexing agents to the electrolyte is essential [108,110].

**Figure 7:**
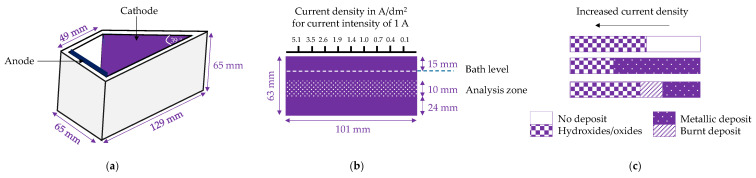
Schemes of: (**a**) Hull cell, (**b**) cathode with analysis zone, (**c**) data interpretation.

**Figure 8:**
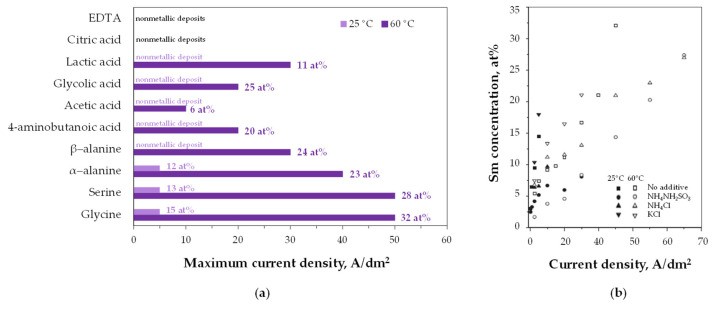
Effect of plating conditions (at 25 and 60 °C) on samarium concentration in Sm–Co alloys: (**a**) complexing agents and current density, (**b**) supporting electrolyte (1M) and current density. Adapted from [114] under License CC BY 4.0.

Wei et al. [109,110,111,112,113,114] investigated Sm–Co alloy electrodeposition using a Hull cell (Figure 7) and parallel electrode setups to identify current density ranges for alloy formation in sulfamate–sulfate baths. They demonstrated [110,112,114] that samarium incorporation and metallic deposit formation depend on the type of complexing ligand, current density/cathode potential, temperature, and the supporting electrolyte (Figure 8), but is only slightly affected by pH in a range of 3–6. At room temperature, only some amino acids (glycine, serine, α-alanine) enabled metallic phase deposition, while (hydroxy)carboxylic acids (acetic, glycolic, lactic) produced no deposits or yielded burnt, powdery layers. Increasing the bath temperature widened the current density range and enhanced samarium incorporation. However, strong chelating complexants (EDTA, citric acid) prevented completely metal deposition. Glycine C_2_H_5_NO_2_ was found to be the most effective complexing agent and this favorable effect was attributed to the stepwise reduction of metal ions from a heteronuclear complex [Co^2+^Sm^3+^(NH_2_CH_2_COO^−^)_3_]^2+^ involving surface-adsorbed H atoms and/or direct electron transfer with the cathode [108,114].

Various electrodeposition modes have been employed for Sm–Co codeposition. The samarium content in the cathodic deposits (Table 4) increases with current density under galvanostatic conditions, with more negative potentials under potentiostatic control [109,110,111,112,113,114,115,116,117,118,119], and with longer duty cycles t_on_/(t_on_ + t_off_) during pulsed current deposition [108,114]. These electrolysis parameters also affect the current efficiency, which in sulfamate–glycine systems ranges from 6% to 32% [108,109,110,111,112,113,114]. However, under identical current density and temperature conditions, direct current was found to promote samarium codeposition and enhance current efficiency compared with pulsed current deposition [113].

Moulin et al. [117] compared the formation of alloys (in a sulfamate–glycine bath) using a Hull cell, 1 mm square molds and micromolding of 5–50 μm patterns. They demonstrated that the scale effect influences both the composition and the deposition rate of the film. Micromolding produced thinner films with lower samarium content, but these films were crack-free, more uniform in thickness, and exhibited good adhesion to the copper seed layer.

**Figure 9:**
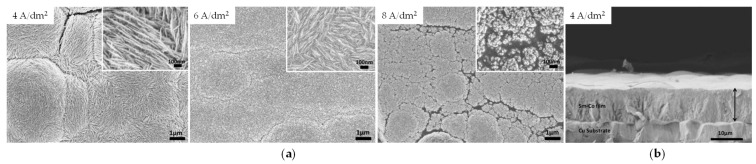
SEM images of Sm–Co alloys produced from sulfamate-glycine solution: (**a**) surface morphologies, (**b**) cross-section. Adapted from [118] under License CC BY 4.0.

The electrodeposited alloys are typically amorphous, regardless of whether they are films (Figure 9) [109,110,111,112,113,114,115,116,117,118,119,120,121] or different nanostructures (nanoparticles, nanowires, nanotubes) [122,123,124]. The reduction in grain size toward a noncrystalline structure was promoted by higher samarium content in the material [114]. Such deposits require subsequent thermal treatment under an inert atmosphere (e.g., Ar at 600 °C [120]) to convert them into crystalline intermetallic compound Sm_2_Co_17_.

The Sm–Co alloys electrodeposited from aqueous solutions may exhibit magnetic properties comparable to those of sputtered materials [113,114]. Magnetic saturation was observed to decrease with increasing samarium content, becoming isotropic in deposits containing more than 30 at% Sm. Park et al. [119] demonstrated that the magnetic properties of Sm–Co alloys varied with different annealing times (0–12 h), influenced by cobalt/Sm_2_Co_17_ ratio, grain size, and porosity, which are governed by the gradual phase transformation from the amorphous phase. The optimal magnetic properties were achieved after 5 h of annealing, yielding a coercivity of 3438 Oe and a saturation magnetization of 81.27 emu/g.

Herrera et al. [123,124] electrodeposited Sm–Co alloys (in a chloride–sulfate bath) into the pores of anodic alumina membranes. Depending on the applied potential, nanowires were formed at less negative potentials (above −0.8 V), whereas more negative potentials (down to −3 V) resulted in the formation of nanotubes, with mixed structures appearing at the intermediate potential of −1 V. These nanostructures exhibited soft ferromagnetic behavior, with the easy magnetization axis aligned parallel to the sample’s major axis and coercivity values below 60 mT. The magnetic hardness was primarily governed by shape anisotropy through saturation polarization (determined by cobalt content), with only a minor influence of geometry (aspect ratio).

#### 3.1.2. Molecular Liquid Solvents

Typical electrolytes based on molecular liquids use DMF [44] or formamide CH_3_NO [125,126,127] as solvents (Table 5). Although both are polar, the solubility of anhydrous metal salts is much lower than in aqueous systems (e.g., a maximum of 0.01M SmCl_3_ in CH_3_NO).

Sato et al. [125,126] used formamide solution with ethylenediamine to obtain films (about 2 μm), while powdery products were produced in the absence of the additive. At low current densities (below 0.8 A/dm^2^) and high samarium concentrations (above 50 at%), the surface exhibited a gray metallic luster, whereas higher current densities (above 1 A/dm^2^) resulted in surface cracking. Within a range of samarium concentrations in the bath from 10 to 50 mol%, the samarium content in the deposits increased from about 0.5% to nearly 90%, which was a much greater extent than expected based on the solution composition. Amorphous layers were formed, and after thermal treatment in an argon atmosphere, only cobalt oxides were identified [126]. Cobalt-rich deposits displayed a high saturation magnetization (180–470 emu/cm_3_) but a lower-than-expected coercive force (below 29 Oe).

Ali et al. [127] used a similar electrolyte to obtain crystalline Sm_2_Co_17_ nanowires within an anodic oxide template. The structural and magnetic properties of the nanowires were examined before and after magnetic field annealing (300 °C, 1 T field applied parallel to the nanowire axis). Annealing enhanced crystallinity and magnetic performance of the nanostructures, increasing coercivity from 91 to 527 Oe for the parallel field and from 101 to 151 Oe for the perpendicular field orientation.

Gao et al. [44] investigated the electrodeposition of Sm–Co alloys in a chloride–LiNO_3_–DMF system for metallurgical applications. The deposits consisted of densely packed spherical structures with a visibly cracked surface. XPS analysis confirmed the presence of metallic cobalt, while samarium occurred in two forms: Sm^3+^ (oxide) and Sm^0^ (metal). The authors proposed a metallurgical strategy for producing a stable bulk alloy comprising four steps: (i) preparation of the electrolyte by dissolving the appropriate salts in the solvent, (ii) continuous electrolysis with a rotating aluminum electrode to convert thin films into bulk material, (iii) demolding of the alloy, and (iv) final smelting.

#### 3.1.3. Ionic Liquid Solvents

Three ionic liquid solvents were initially tested to evaluate their suitability for producing Sm–Co alloys (Table 5), both as thin films [65,66] and as nanostructures [128,129,130]. These included [BMP][NTf_2_] [65,128], [BMP][DCA] [66], and [EMI][Cl] [129,130].

Ispas et al. [65] used potentiostatic square pulses to codeposit elements typically confined to distinct potential regions in [BMP][NTf_2_] (120 °C). The resulting Sm–Co layer was cracked, with cobalt-rich centers and samarium-rich edges. Similar oxygen levels (41–45 at%) suggested that this uneven metal distribution arose from nonuniform current across the electrode. Subsequent studies [128] showed that SmCo_7_ nanoparticles could be obtained by potentiostatic deposition at room temperature from the same electrolyte. As the disproportionation reaction (4) can occur and Sm^2+^ can act as a reducing agent, the following mechanism was proposed to explain the observed formation of intermetallics:

17Sm^2+^ + 7Co^2+^ → 16Sm^3+^ + SmCo_7_ (16)

Consequently, the following total electrode reaction was proposed as feasible at −1.6 V:

Sm^3+^ + 7Co^2+^ + 17e → SmCo_7_ (17)

Gong et al. [129] synthesized SmCo nanoparticles using a similar potentiostatic approach in 1-methyl-3-ethylimidazolium chloride [EMI][Cl], with the aim of subsequently converting them into PtSmCo nanoparticles as electrocatalysts for enhanced oxygen reduction. However, these SmCo nanoparticles were only characterized by SEM morphologies, without further structural or compositional analysis. The [EMI][Cl] ionic liquid was also demonstrated as a suitable solvent for the electrochemical preparation of SmCo nanowires without a template [130]. The addition of SmCl_3_ to the CoCl_2_–[EMI][Cl] electrolyte shifted the Co^2+^ reduction potential (by +0.2 V) and increased the current density. The metal content, diameter, and length of the nanowires could be readily tuned by varying the deposition conditions. The smallest SmCo nanowire diameters achieved were 50–60 nm, substantially smaller than the 500 nm diameter of Co nanowires. It was found that the nanowire diameter decreases with lower temperature and shorter deposition time, crystallinity improves with increased SmCl_3_ concentration, and the Sm:Co ratio in the alloy is influenced by the applied potential and bath composition. This method is particularly notable for significantly simplifying the electrochemical preparation of nanowires.

Molodkina et al. [66] investigated alloy codeposition from the [BMP][DCA] ionic liquid and observed samarium codeposition at potentials more positive (by +0.4 V) than in a single-component Sm^3+^ bath. The analysis showed that an increase in water concentration in the solution significantly inhibited Sm–Co codeposition. It was attributed to the formation of an oxide film on the deposit surface, which prevented further reduction of cobalt ions and thus hindered alloy codeposition.

#### 3.1.4. Deep Eutectic Solvents

Choline chloride-based systems [77,78,131] and urea-acetamide-alkali bromide mixtures [79,80] have been investigated (Table 5). Gómez et al. [77] observed a significant enhancement in the stirring effect on Sm–Co codeposition from the ChCl–U eutectic mixture under both galvanostatic and potentiostatic conditions. The composition of the deposits was dependent on the substrate material, with samarium-richer layers (80 wt% Sm) produced on Ni/Cu/Au slides, compared to glassy carbon substrates (27–75 wt% Sm). Notably, at more negative potentials and extended deposition times, stable high levels of samarium were consistently observed in the deposits. Although the effect was not explored in detail, it was evident that ion transport played a crucial role. The deposits exhibited a nodular morphology, with a tendency to form finer grains as the deposition time became more negative. Unlike pure samarium deposits, no cracking was reported.

**Figure 10:**
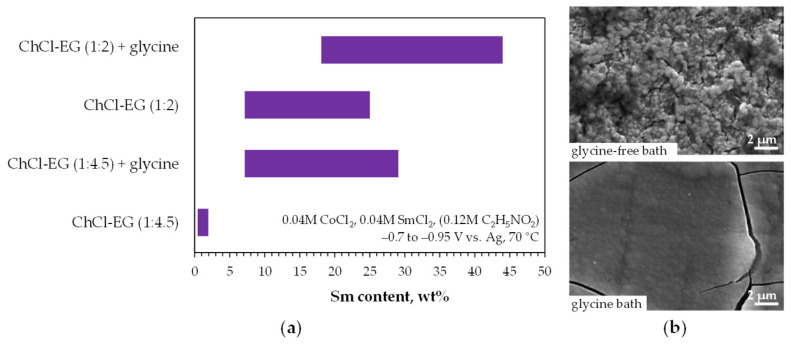
Effect of chloride–ChCl–EG bath composition on: (**a**) Sm concentration in deposits, (**b**) surface morphology of 20%Sm–Co alloys. Adapted from [78] under License CC BY 4.0.

Panzeri et al. [78,131] investigated the effect of glycine addition on the potentiostatic electrodeposition of alloys from metal chloride–ChCl–EG mixtures with varying compositions. Higher samarium incorporation was observed at lower ethylene glycol concentrations and in the presence of glycine (Figure 10a). The addition of glycine promoted also film growth by increasing the maximum available layer thickness of smooth surfaces, despite some cracking (Figure 10b). Interestingly, in this electrolyte, an increased stirring rate reduced samarium content in the alloys (from 12–13 wt% at 10 rpm to 7–8 wt% at 200 rpm at −0.8 V vs. Ag) [131]. Although only the hcp-Co phase was identified in the deposits [78], it was assumed that a cobalt metal matrix with samarium atoms was formed. Indeed, annealing of the deposits led to the formation of Sm_2_Co_17_ intermetallics [131]. The produced alloys were ferromagnetic, with improved in-plane coercive fields. The coercivity of the Co-20%Sm alloy was higher when produced from the glycine-free bath (270 Oe vs. 100 Oe), and significantly higher than that of pure cobalt (73 Oe) [78].

In the urea-acetamide-bromide melts [79,80], samarium electroreduction is induced in the presence of cobalt ions, although it does not deposit from a single-metal bath. Under such conditions, either amorphous nanoparticles or dense and smooth films containing 8–32 wt% Sm [80] are formed, which transform into crystalline Sm_2_Co_17_ [80] or SmCo_5_ [79] phases after annealing. The coercive field of the as-deposited materials depended on the samarium concentration and measurement temperature, while annealing significantly reduced their magnetic properties [80].

#### 3.1.5. Molten Salt Electrolytes

Molten salt electrolytes are rather seldom used for the electrosynthesis of Sm–Co alloys; however, the chloride system (LiCl–KCl eutectic) has been investigated on inert electrodes (W, Mo, Cu) [132,133,134]. As in low-temperature electrolytes, the presence of cobalt ions induces the co-reduction of samarium ions at more positive potentials than the reduction of lithium ions due to formation of intermetallic compound. The letter are produced through reaction (6), preceded by reduction of Co^2+^ to metal and Sm^3+^ to Sm^2+^. However, when the cathodic potential is forced to values more negative than the Li/Li^+^ pair, the reduction of Sm^2+^ by liquid lithium can take place [133]:

2Sm^2+^ + 2Co + Li → SmCo_2_ + 2Li^+^ (18)

**Figure 11:**
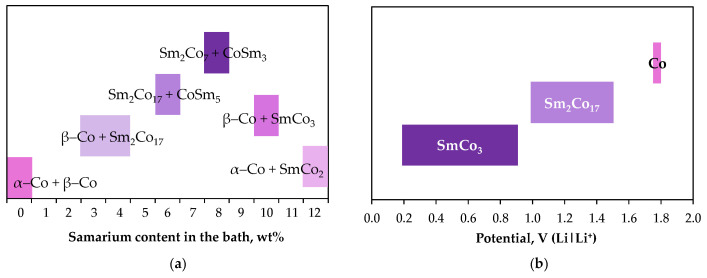
Effect of plating parameters on phase composition of the Sm–Co alloys produced from SmCl_3_–CoCl_3_–LiCl–KCl melts on inert substrates: (**a**) GD: 594 A/dm^2^, 700 °C (adapted from [132]), (**b**) PD: 450 °C (adapted from [134]).

In contrast, high operating temperatures result in the formation of thick layers (up to 80 µm [133]), though these layers are not dense. They are typically composed of grains with distinct boundaries, often hexagonal in shape [132,134], and are made up of intermetallic phases (Table 5). Liu et al. [132] demonstrated that as the mass percentage of SmCl_3_ increased from 0 to 12 wt.% during galvanostatic deposition, the intermetallic phases shifted from the Co-rich to the Sm-rich side (Figure 11a). These intermetallic compounds were observed as multilayer planar structures. Additionally, Iida et al. [134] showed that phases with different stoichiometry can form on the cathode by shifting the electrode potential (Figure 11b).

Magnetic properties were found to vary depending on the crystallographic structure of the intermetallic compound, with increasing samarium content [132]. The Sm_2_Co_17_ and SmCo_5_ intermetallic compounds exhibited relatively high magnetic densities, reaching up to 157 emu/g and 145 emu/g, respectively.

### 3.2. Sm–Ni–(Fe) Alloys

Electrodeposited samarium binary and ternary alloys with other iron-group metals (nickel and iron) have also been developed [135,136,137,138,139,140]. Although the available data on this topic are considerably scarcer than for the Sm–Co system, the potential of electrochemical synthesis in some electrolytes has been clearly demonstrated (Table 6).

Murali Krishna et al. [135] synthesized Sm–Ni alloys using a deep eutectic solvent electrolyte. The obtained coatings exhibited a smooth surface with a metallic luster; however, both metallic and samarium oxide phases with mixed oxidation states (+2, +3) were identified. These deposits were developed for unconventional applications as catalysts for electrochemical hydrogen evolution. Remarkably, the materials demonstrated superior long-term electroactivity (50 h) compared to nickel in an alkaline medium (1M KOH), with the overpotential shifted by approximately 0.2 V toward more positive values (at 1 A/dm^2^).

In turn, Li et al. [136] obtained Sm–Ni alloys from a SmCl_3_–NiCl_2_–LiCl–KCl melt using an inert substrate. They confirmed the promoting effect of nickel species on samarium electroreduction, which resulted in deposits containing a mixture of intermetallic phases. The magnetic properties of the SmNi_x_ phases were found to depend on their stoichiometry, exhibiting different magnetic saturation values (138 emu/g for SmNi_5_, 63 emu/g for SmNi_2_, and 53 emu/g for SmNi), higher than that of pure nickel (46 emu/g).

Sulfamate–glycine aqueous solutions have proven to be highly effective for the electrodeposition of thick Sm–Fe alloys (up to 20 μm) [137,138]. Kou et al. [137] reported pronounced changes in alloy morphology as a function of current density. At low current densities (6–10 A/dm^2^), the films exhibited coarse grains and relatively rough surfaces. With increasing current density, the grains became finer and the deposits more compact, and at medium current densities (20–30 A/dm^2^), the films showed a silver-gray metallic luster, a relatively smooth surface, and only minor cracking. However, when the current density was further increased (above 30 A/dm^2^), the surface became rougher again, developing a spongy, porous structure. The application of an external magnetic field during electrodeposition influenced the growth of the metallic phase, resulting in finer-grained films, with this effect being more pronounced in the perpendicular configuration than in the parallel one. Simultaneously, increasing the magnetic field intensity in the parallel configuration suppressed samarium codeposition, decreasing the samarium content in the deposits (from 4.5 at% at 0 T to 3 at% at 4 T), whereas no such effect was observed in the perpendicular configuration. In the absence of a magnetic field, the Sm–Fe film consisted of pure iron and SmFe_12_ [137] or Sm_2_Fe_17_ [138], but with increased current density [137] or while under an applied magnetic field oxide phase Sm_3_Fe_5_O_12_ also appeared under certain conditions.

Li et al. [139] obtained amorphous Sm–Fe alloys from a urea–AT–bromide solvent and observed that the deposit roughness increased with higher samarium content. They also noted that, under the same deposition conditions, the samarium content was higher in Sm–Fe than in Sm–Co alloys, despite iron being a less noble metal than cobalt. A clear anisotropy was observed, with the easy magnetization direction lying in the plane of the film.

Gandi et al. [140] reported the galvanostatic electrodeposition of ternary Sm–Ni–Fe alloys from aqueous sulfate baths. They found that, although individual samarium phases were not detected, an increase in the samarium content in the alloys was accompanied by a decrease in the content of the other two metals, an increase in grain size (from 71 nm for 0% Sm to 156 nm at 25 at% Sm), a reduction in lattice strain (from 0.23% for 0% Sm to 0.14% at 25 at% Sm), and an increase in anisotropy field values.

### 3.3. Other Alloys

Other samarium alloys are typically electrodeposited from molten salt systems, primarily as a method for obtaining the material itself or for its recovery from nuclear waste. In such processes, the extraction and/or separation of samarium from other lanthanides in molten metal salt baths is facilitated by the addition of metal compounds that promote the formation of intermetallic phases. Commonly, the following additives have been tested using inert electrodes: (i) aluminum salts, leading to the formation of SmAl_x_ intermetallics (e.g., AlF_3_ in SmF_3_–LiF–CaF_2_ [99] and AlCl_3_ in Sm_2_O_3_–LiCl–KCl [141]); and (ii) zinc chloride, resulting in the formation of SmZn_x_ intermetallics in SmCl_3_–LiCl–KCl systems [142,143].

Alternatively, molten salt systems have also been explored as media for alloy fabrication. Liu et al. [144] developed a process for preparing dendritic Sm_x_Cu_y_ intermetallic compounds as potential catalysts for chemical industry. In turn, Wei et al. [145] synthesized various phases of Mg–Li–Sm alloys by molten salt electrolysis in SmCl_3_–MgCl_2_–LiCl–KCl melts, demonstrating that the best corrosion resistance was achieved for alloys containing 0.7 wt% samarium, whereas both lower and higher samarium contents (up to 3.7 wt%) led to a deterioration in the corrosion resistance of Mg–Li alloys.

Notably, the modification of molten baths with additional alloying metal salt leads to the formation of intermetallic phases with samarium on inert substrates, either the same as [95,99] or different from those produced on reactive electrodes (Table 4). For example, SmCu and SmCu_5_ (on Mo substrate) were formed [144] in addition to SmCu_6_ (on a Cu substrate) [88], SmZn_12_ instead of SmZn_17_ on a liquid zinc substrate [143], SmAl_3_ and SmAl_4_ [141] instead of SmAl_2_ and SmAl_3_ (both on Al substrate) [89]. The current efficiency of samarium extraction at the cathode depends on the electrolysis mode [141] and ranges from 89–94% (in the presence of aluminum chloride) [141] to nearly 100% (in the presence of zinc chloride) [142].

## 4. Electrodeposition of Samarium Oxides

The significant hindrance to reducing samarium ions to the elemental state presents a challenge for the formation of metallic phases. However, it also opens up new opportunities for the deposition of samarium compounds, e.g., hydroxide Sm(OH)_3_ [37], monosulfide SmS [146], telluride SmTe [147], selenide SmSe_x_ [148], alkali hexacyanoferrates M[SmFe(CN)_6_] [149], acetates Sm_2_O_3_∙xCO_2_ [150]. These compounds are formed as thin layers through secondary chemical reactions at the cathode surface, preceded by a change in the ionic composition of the electrolyte near the electrode resulting from the reduction of species from the bath.

Preferential hydrogen evolution at the cathode in diluted aqueous solutions of samarium(III) salts (e.g., nitrate, sulfate):

2H_2_O + 2e → H_2_ + 2OH^−^ (19)

results in rapid alkalization of the adjacent electrolyte. Morrison et al. [37] found that, as the electrode voltage was varied, the pH of a samarium(III) nitrate-ammonium acetate solution at the cathode surface increased from an initial value of approximately 5 at 0 V to nearly 12 at around −1.2 V. This pH change induces the precipitation of samarium(III) hydroxide, Sm(OH)_3_:

Sm^3+^ + 3OH^−^ → Sm(OH)_3_ (20)

Ruiz et al. [151] reported that under oxygen-saturated conditions, the mass of the deposit formed on the cathode in a SmCl_2_ solution (10 mM, pH 5.8) was greater than that under nitrogen-degassed conditions. Thus, they proposed that dissolved oxygen facilitates the formation of hydroxide ions:

O_2_ + 2H_2_O + 4e → H_2_ + 4OH^−^ (21)

and, in consequence, the precipitation of hydroxide (20), which subsequently transforms into oxide under temperature [152,153]:

2Sm(OH)_3_ → Sm_2_O_3_ + 3H_2_O (22)

Notably, varying the oxygen saturation of the solution resulted in deposits with slightly different morphologies.

This simple approach (20) to the precipitation of hydroxide/oxide films was questioned by López et al. [34], who observed partial reduction of Sm^3+^ ions to Sm^2+^ during cyclic voltammetric measurements. Additionally, they identified Sm(II) and Sm(III) oxide-bonded species incorporated into the co-deposited nickel layer. As a result, they suggested that the mechanism of compound formation should involve an additional reaction:

Sm^2+^ + 2OH^−^ → Sm(OH)_2_ (23)

Wei et al. [110] conducted a series of experiments in a Hull cell using a samarium sulfamate solution. They observed two distinct regions: the formation of hydroxide/oxide deposits or no deposition at all, with inorganic compounds forming at higher current densities (Figure 7c). This region narrowed to higher current densities (above 16 A/dm^2^) with increasing temperature and in the presence of glycine.

Electrodeposition accompanied by precipitation of samarium hydroxides on the cathode surface has shown a variety of applications in the formation of different types of materials. These include: (i) Sm_2_O_3_ nanoparticles [153], (ii) Sm_2_O_3_ targets for nuclear applications [37], (iii) Sm_2_O_3_ and Sm-doped cerium oxide (ceria) as conversion coatings for anticorrosion protection [151], as solid electrolyte in fuel cells (due to improved ionic conductivity by highest formation of oxygen vacancies in Ce_1−x_Sm_x_O_2−x/2_) [152] or luminescent thin films (red-orange spectral range) [154], (iv) luminescent samaria–zinc oxide multilayers for solid state lighting (550 nm) [155], (v) luminescent Sm-doped copper(I) oxide for photodiode and photovoltaic purposes [156], (vi) samarium cobalt oxide nanoparticles for electrochemical capacitors [157], (vii) protective composite coatings [34,158], etc.

## 5. Conclusions

Electrodeposition is a widely employed, scalable, and relatively straightforward technique for fabricating metallic or inorganic coatings and nanostructures, utilizing both aqueous and non-aqueous electrolytes (Table 7). The method offers significant flexibility, enabling precise control over the composition, structure, and morphology of the deposited layers through adjustments in electrolyte composition and deposition parameters. This adaptability makes electrodeposition an attractive option for producing advanced materials. Furthermore, it serves as an effective technique for the selective recovery of metals during electrometallurgical processing, particularly on an industrial scale, offering both economic and environmental benefits by recovering valuable metals, also from waste streams.

**Figure 12:**
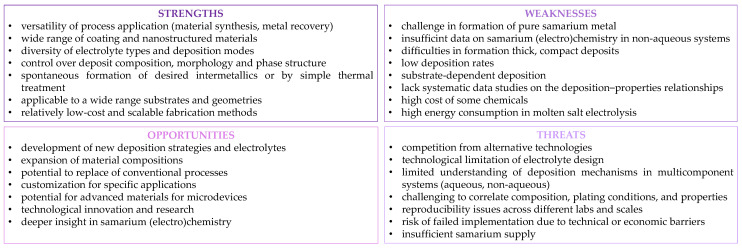
Samarium electrodeposition—SWOT analysis.

Despite its potential, electrodeposition faces significant difficulties when applied to samarium and samarium-based materials (Figure 12). One of the primary obstacles is the difficulty in obtaining pure samarium metal from traditional aqueous solutions or molten salts. This limitation necessitates the exploration of alternative electrolytes, such as molecular liquids, ionic liquids or deep eutectic solvents, along with the optimization of deposition parameters, including the selection of bath components (additives), current/potential conditions, and temperature. Moreover, samarium-containing deposits exhibit typically inhomogeneous microstructures, low deposition rates, and poor current efficiency.

Paradoxically, these challenges open up new opportunities for research and scientific exploration. Untested deposition strategies, such as varying current/potential modes, alongside the development of novel electrolyte formulations, present promising solutions for achieving more consistent and reproducible results. The development of reaction mechanisms, with a focus on the spontaneous formation of desirable intermetallic phases, is crucial for their usage for magnetic purposes. Moreover, optimizing the electrodeposition process could lead to more energy-efficient methods for recovering samarium from other rare-earth elements, thus addressing the growing demand for these critical materials. With continued research and technological advancements, the electrodeposition of samarium could become a key aspect of sustainable metal recovery and the development of advanced coatings for a diverse range of industrial applications.

## Figures and Tables

**Table 1:** Standard electrode potentials (vs. SHE) of half-reactions of samarium species (25 °C, 1013.25 hPa). Adapted from [38].

Electrode Equilibrium *	Standard Potential E°, V
Acid solution (pH = 0)	
Sm^3+^|Sm^2+^	−1.33
SmOH^2+^, H^+^|Sm_(c)_	−2.15
Sm^3+^|Sm_(c)_	−2.30
Sm^2+^|Sm_(c)_	−2.68
Alkaline solution (pH = 13.996)	
Sm(OH)_3(pt)_ |Sm_(c)_, OH^−^	−2.75
Sm(OH)_2_∙H_2_O_(c)_|Sm_(c)_, OH^−^	−2.77
Sm(OH)_3(c)_|Sm_(c)_, OH^−^	−2.78
Sm(OH)_3(c)_|Sm(OH)_2_∙H_2_O_(c)_, OH^−^	−2.80

* c—pure crystalline solid, pt—hydrous precipitate (amorphous solid with variable water content).

**Table 2:** Electroreduction of samarium species from ionic liquid-based electrolytes for potential applications.

Electrolyte Composition	Electrolysis Conditions	Metal Formation (Evidence Method)	Application Context	Ref.
Electrodeposition				
0.06M Sm(OTf)_3_ in [BMP][NTf_2_]	PD: −3.1 V vs. Ag/Ag^+^, 25 °C, 5 h, Cu substrate	yes (EDS)	metal electrowinning	[52]
0.06M Sm(OTf)_3_ in [Me_3_NBu][NTf_2_]				
0.01M Sm(NTf_2_)_3_ in [BMP][NTf_2_]	PD: −1.6 or −2.5 V vs. Ag/Ag^+^, 100 °C, 3C, GC substrate	yes (EDS, TEM)	metal recovery	[56]
0.4M Sm(NTf_2_)_3_ in TMP	PD: −3.2/−2.5 V vs. Pt, 30 °C, 3 h, GC/Cu substrate	yes (EDS, XPS)	magnetic materials	[57]
0.1M Sm(OTf)_3_ or Sm(NO_3_)_3_∙H_2_O or SmCl_3_ in [BMP][DCA]	PD: −2.0 to −2.8 V vs. Ag/Ag^+^, 25–60 °C, 4 h, GC/Ni substrate	yes (XPS)	metal recovery	[58]
0.5M Sm(NTf_2_)_3_ in [BMP][NTf_2_]	PD: −2.5 V vs. Ag/Ag^+^, 100 °C, 5 h, Cu substrate	yes (XPS)	metal recovery	[59]
0.1M Sm(OTf)_3_ or Sm(NO_3_)_3_∙H_2_O in DMI	PD: −3.0 V vs. Ag/Ag^+^, 70 °C, 5 h, Cu substrate	yes (XPS)	metal electrowinning	[60]
0.1M Sm(NTf_2_)_3_ in [BMP][NTf_2_]	PD: −2.6 V vs. Ag/Ag^+^ or GD: 0.01 A/dm^2^ 120 °C, 1.1C, Au substrate	yes (XRD)	magnetic materials	[65]
0.01M Sm(OTf)_3_ in [BMP][DCA]	PD: −1.8 V vs. Ag/AgCl, 24 °C, 1 h, Pt substrate	yes (XPS)	magnetic materials	[66]
Cyclic Voltammetry				
0.03M Sm(NTf_2_)_3_ in [BMP][DCA]	−0.35 to −2.3 V vs. Fc/Fc^+^, 100 mV/s, 25 °C, Pt substrate	possible (CV)	selective separation	[53]
0.22M Sm(NTf_2_)_3_(H_2_O)_3_ in [Me_3_NBu][NTf_2_]	1 to −3.2 V vs. Ag, 100 mV/s, 25 °C, Pt substrate	possible (CV)	selective separation in nuclear industry	[54]
0.1M Sm(NTf_2_)_3_ in [BMP][NTf_2_]	0 to −2.5 V vs. Ag, 10 mV/s, 25 °C, GC substrate	no (CV)	redox batteries	[55]
0.03M Sm^3+^ in [Me_3_NBu][NTf_2_]	1.5 to −2.5 V vs. Ag/Ag^+^, 25 °C, Pt substrate	possible (visual)	selective separation in nuclear industry	[62]

**Table 3:** Electroreduction of samarium ions from deep eutectic solvents.

Electrolyte Composition	Electrolysis Conditions	Metal Formation (Evidence Method)	Ref.
0.045M Sm(NO_3_)_3_ in ChCl–U (1:2)	PD: −1.9 V vs. Ag/AgCl, 70 °C, 0.5 h, GC substrate	yes (SEM)	[77]
0.04M or 0.08M SmCl_3_∙6H_2_O in ChCl–EG (1:2 or 1:4.5)	CV: 0.5 to −1.5 V vs. Ag, 70 °C, 10 mV/s, Pt substrate	no (CV)	[78]
0.1M SmCl_3_ in U–AT(34%)–NaBr(14.5%)–KBr(1.5%) *	CV: 0.85 to −1.0 V vs. Ag/AgCl, 80 °C, Pt substrate	no (CV)	[79]
	PD: −0.6 to −1.0 V vs. Ag/AgCl, 80 °C, Pt substrate	no (visual)	
0.1M SmCl_3_ in U–AT(50%)–NaBr(15%) *	CV: 0.6 to −1.4 V vs. Ag, 70 °C, 100 mV/s, Pt substrate	no (CV)	[80]

* AT—acetamide CH_3_–CO–NH_2_.

**Table 4:** Electroreduction of samarium species from molten salt electrolytes.

Electrolyte Composition	Electrolysis Conditions	Cathode Substrate	Metal Formation (Evidence Method)	Ref.
Chloride Systems				
0.1M SmCl_3_ in LiCl–KCl eutectic	CV: 0 to −2.5 V vs. Ag/AgCl, 530 °C, 100 mV/s	Mo ^1^	no (CV)	[83]
	CV: 0 to −2.5 V vs. Ag/AgCl, 530–600 °C, 100 mV/s	Al ^2^	yes: Sm_x_Al_y_ (OCP)	
1 wt% SmCl_3_ in LiCl–KCl eutectic	PD: −2.4 V vs. Ag/Ag^+^, 500 °C, 3 h	Cu ^2^	yes: Sm_x_Cu_y_ (EDS, CV)	[84]
	PD: −1.9 or −2.0 V vs. Ag/Ag^+^, 500 °C, 0.5 h	Al ^2^	yes: Sm_x_Al_y_ (EDS, CV)	
0.07M SmCl_3_ in LiCl–KCl eutectic	PP: −1.4 to –0.4 V vs. Ag/AgCl, 500 °C, 15 h	PbBi_liq_ ^2^	yes: SmBi (XRD, CV)	[85]
0.1M SmCl_3_ in NaCl–2CsCl eutectic	CV: 0.7 to −2.5 V vs. Ag/AgCl, 550–650 °C, 50–300 mV/s	Mo ^1^	no (CV)	[86]
	PD: −1.75 V vs. Ag/Ag^+^, 550 °C, 8 h	Ga_liq_ ^2^	yes: SmGa_6_ (XRD, EDS)	
min. 0.03M SmCl_3_ in KCl	CV: 0.2 to −1.7 V vs. Ag/AgCl, 815 °C, 100 mV/s	Au	possible (CV)	[87]
min. 0.03M SmCl_3_ in LiCl–KCl–(KF)	CV: 0.2 to −2.8 V vs. Ag/AgCl, 550 °C, 25 mV/s	W ^1^	no (CV)	
0.1M SmCl_3_ in LiCl–KCl eutectic	PD: −2.2 V vs. Ag/AgCl, 450 °C, 3 h	Al ^2^	yes: SmAl_3_, SmAl_2_ (EDS, XRD)	[89]
0.001M SmCl_3_ in NaCl–CaCl_2_ eqmol melt	CV: 1.2 to −2.6 V vs. Ag/Ag^+^, 550 °C, 20 mV/s	W ^1^	no (CV)	[92]
0.1M SmCl_3_ in LiCl–KCl eutectic	PD: −1.6 or −2.0 V vs. Ag/AgCl, 500 °C, 20 h	SnIn_liq_ ^2^	yes: SmSn_2_, Sm(In_1.5_ Sn_1.5_) (XRD, EDS, CV)	[93]
1wt% SmCl_3_ in LiCl–KCl eutectic	PD: −1.6 V vs. Ag/AgCl, 500 °C, 10 h	Zn_liq_ ^2^	yes: Sm_2_Zn_17_ (XRD, SEM)	[95]
	GD: 15 A/dm^2^, 500 °C, 8 h			
0.5 mol SmCl_3_ in LiCl–KCl eutectic	PD: 0.1 V vs. Li/Li^+^, 500 °C, 72 h	Ni ^2^	yes: SmNi_2_ (XRD)	[101]
	GD: 5 A/dm^2^, 500 °C, 1 h			
0.5mol% SmCl_3_ in LiCl–KCl eutectic	GD: 5 A/dm^2^, 450 °C, 24 h	Co ^2^	yes: SmCo_2_ (XRD)	[103,104]
	PD: 0.2 V vs. Li/Li^+^, 450 °C, 1 h;			
Fluoride Systems				
0.5M SmF_3_ in LiF–CaF_2_ eutectic	CV: 0.4 to −2.0 V vs. Pt, 850 °C, 100 mV/s	Mo ^1^	no (CV)	[82]
	GD: 200 A/dm^2^, 850 °C, 1 h	Ni ^2^	yes: Sm_x_Ni_y_ (EDS, SEM)	
0.094M SmF_3_ in 2LiF–BeF_2_ eutectic	CV: 0.8 to −0.15 V vs. Be/Be^2+^, 600 °C	W ^1^	no (CV)	[88]
	PD: −0.14 V vs. Be/Be^2+^, 600 °C, 1 h	Cu ^2^	yes: SmCu_6_ (XRD)	
	PD: −0.14 V vs. Be/Be^2+^, 600 °C, 1 h	Ni ^2^	yes: Sm_2_Ni_17_ (XRD)	
	PD: −0.07 V vs. Be/Be^2+^, 600 °C, 1 h	Al ^2^	yes: SmAl_4_ (XRD)	
0.001M SmF_3_ in 2LiF–BeF_2_ eutectic	CV: 0.2 to −1.7 V vs. Pt, 540 °C, 50 mV/s	Mo ^1^	no (CV)	[90,91]
Chloride–Fluoride Systems				
0.1M SmCl in LiF–CaF_2_ eutectic	GD: 200 A/dm^2^, 1100 °C, 1 h	Fe ^2^	yes: Sm_2_Fe_17_ (XRD)	[105]

^1^ Inert cathode. ^2^ Reactive cathode.

**Table 5:** Electrodeposition of Sm–Co alloys from various electrolytes.

Electrolyte Composition	Electrolysis Conditions	Alloy Deposit	Ref.
Aqueous Solutions			
0.9M Sm(NH_2_SO_3_)_3_, 0.12M Co(NH_2_SO_3_)_2_, 0.36M C_2_H_5_NO_2_, 1M NH_4_NH_2_SO_3_; pH 7	GD: 20–45 A/dm^2^, 23 °C	3–8 at% Sm; film	[108]
	PCD: 40 A/dm^2^, 10 Hz, t_on_/(t_on_ + t_off_) 0.1–1, 23 °C	3–7 at% Sm; film	
1M Sm(NH_2_SO_3_)_3_, 0.05M CoSO_4_, 0.15M C_2_H_5_NO_2_, 1M NH_4_NH_2_SO_3_; pH 5.2	GD: 35–70 A/dm^2^, 50 C, 25 or 60 °C	3–8 at% Sm; film: amorphous or Sm_2_Co_17_	[109]
0.05M Sm_2_O_3_, 0.3M HNH_2_SO_3_, 0.07M CoSO_4_, 0.21M C_2_H_5_NO_2_, 1M NH_4_NH_2_SO_3_; pH 6	GD: 2.8–6 A/dm^2^, 60 °C, 0.3 h	2–9 at% Sm; amorphous film, Sm_2_Co_17_ after reduction-diffusion	[119]
Sm(NH_2_SO_3_)_3_, 0.1M CoSO_4_, 0.3M C_2_H_5_NO_2_, 0.5M H_3_BO_3_; pH 2.5	PD: −1.6 to −2.1 V vs. Ag/AgCl, 5 or 35 °C	5–40 at% Sm; film, amorphous: Sm_2_Co_17_ after annealing	[120]
0.2M SmCl_3_, 0.1M CoCl_2_, 0.7M H_3_BO_3_, 0.2M C_2_H_5_NO_2_, 0.05M C_6_H_8_O_6_, 1M HCl; pH 2	DCV: 2.5 V	3 at% Sm; amorphous nanowires, Sm_2_Co_17_ after annealing	[122]
0.06M SmCl_3_, 0.06M CoSO_4_, 0.5M H_3_BO_3_; pH 3	PD: −0.8 to −3.0 V vs. Ag/AgCl, 27 °C, 0.3 h	10–50 at% Sm; amorphous nanowires or nanotubes	[123,124]
Molecular Liquid Solvents			
0.05M SmCl_3_, 0.075M CoCl_2_, 0.015M LiNO_3_ in DMF	PD: −2.8 vs. Ag, 50 °C, 0.5 h	16 at% Sm; film	[44]
SmCl_3_, CoCl_2_, ethylenediamine in CH_3_NO	GD: 1 A/dm^2^, 25 °C, 1 h	21% Sm, amorphous	[125,126]
SmCl_3_, CoCl_2_ in CH_3_NO	GD: 25 °C	nanowires, Sm_2_Co_17_	[127]
Ionic Liquid Solvents			
0.05M Sm(NTf_2_)_3_, 0.05M Co(NTf_2_)_2_ in [BMP][NTf_2_]	PPD: −0.8 V 1s/ −2 V 1s vs. Pt, 120 °C, 2 h	21/41 at% Sm; film	[65]
0.01M Sm(OTf)_3_, 0.01M CoCl_2_, in [BMP][DCA]	PD: −1.8 V vs. Ag/AgCl, 24 °C, 1 h	film (XPS)	[66]
0.005M Sm(NTf_2_)_3_, 0.03M Co(NTf_2_)_2_ in [BMP][NTf_2_]	PD: −1.5 to −2.5 V vs. Ag/Ag^+^, 25 °C, 1.2 C	SmCo_7_ nanoparticles	[128]
1.25–2.5mol% SmCl_3_, 60%mol CoCl_2_ in [EMI][Cl]	PD: −0.6 to −0.7 V vs. Ag/Ag^+^, 120 °C, 0.1–0.3 h	Co:Sm 2–35; nanowires	[130]
Deep Eutectic Solvents			
0.04M SmCl_3_∙6H_2_O, 0.04MCoCl_2_∙6H_2_O, (0.12M C_2_H_5_NO_2_) in ChCl–EG	PD: −0.7 to −0.97 V vs. Ag, 70 °C	0.5–44 wt% Sm; film; Sm_2_Co_17_ after annealing	[78,131]
0.045M Sm(NO_3_)_3_, 0.018M CoCl_2_ in ChCl–U	PD: −1.6 to −1.9 V vs. Ag/AgCl, 70 °C, 0.25–0.7 h	27–75 wt% Sm; film	[77]
0.1M SmCl_3_, 0.04M CoCl_2_ in U–AT(34%)–NaBr(14.5%)–KBr(1.5%)	PD: −0.7 to −0.9 V vs. Ag/AgCl, 80 °C, 0.5 h	up to 50 wt% Sm; amorphous film, after annealing: SmCo_5_, Sm_2_Co_7_, Sm_2_Co_17_	[79]
0.1M SmCl_3_, 0.2M CoCl_2_ in U–AT(50%)–NaBr(15%)	PD: −1.15 to −1.35 V vs. Ag, 70 °C	8–79 wt% Sm; amorphous film, Sm_2_Co_17_ after annealing	[80]
Molten Salt Electrolytes			
2–12 wt% SmCl_3_, 0.5–3 wt% CoCl_2_ in LiCl–KCl eutectic	GD: 594 A/dm^2^, 700 °C, 2 h; Mo substrate	22–33 wt% Sm; flaky: Sm_2_Co_17_, SmCo_5_, Sm_2_Co_7_, SmCo_3_	[132]
2–12 wt% SmCl_3_, 0.5–3 wt% CoCl_2_ in LiCl–KCl eutectic	GD: 30 A/dm^2^, 550–750 °C; PD: −2.7 V vs. Ag; 0.5 h; W substrate	film: SmCo_2_	[133]
0.5mol% SmCl_3_, 0.1 mol% CoCl_2_ in LiCl–KCl eutectic	PD: 0.2 to 1.5 V vs. Li/Li^+^, 450 °C, 1 h; Cu substrate	nonuniform layer: Sm_2_Co_17_, SmCo_3_	[134]

**Table 6:** Electrodeposition of Sm–Ni, Sm–Fe and Sm–Ni–Fe alloys from various electrolytes.

Electrolyte Composition	Electrolysis Conditions	Deposit	Application	Ref.
Sm–Ni Alloys				
0.2M SmCl_3_, 0.05M NiCl_2_, 0.12M C_2_H_5_NO_2_ in ChCl–EG eutectic	GD: 0.1–0.5 A/dm^2^, 70 °C, 2 h	2–6 wt% Sm; metallic with oxides	hydrogen evolution catalyst	[135]
2 wt% SmCl_3_, 2 wt% NiCl_2_ in LiCl–KCl eutectic	PD: −1.5 to −2.2 V vs. Ag/Ag^+^, 700 °C, 6 h; Mo substrate	19–55 wt% Sm; SmNi_5_, SmNi_2_, SmNi, SmOCl	magnetic materials	[136]
Sm–Fe Alloys				
0.6M SmCl_3_, 0.1M FeCl_2_, 0.5M H_3_BO_3_, 0.06M HNH_2_SO_3_, 0.21M C_2_H_5_NO_2_, (0.4M NaCl); pH 3	GD: 0.2–35 A/dm^2^, 25 °C, 0.2 h; (magnetic field)	0.5–8 at% Sm; SmFe_12_, Sm_3_Fe_5_O_12_	magnetic materials	[137,138]
0.1M SmCl_3_, 0.04M FeCl_2_ in U–AT(50%)–NaBr(14%)–KBr(2%)	PD: −0.9 to −1.2 V vs. Ag/Ag^+^, 80 °C, 0.4 h	Sm_x_Fe x = 0.05–0.4; amorphous	magnetic materials	[139]
Sm–Ni–Fe Alloys				
0.007–0.03M Sm_2_(SO_4_)_3_, 1M NiSO_4_, 0.03M FeSO_4_, 0.8 g/L H_3_BO_3_	GD: 1 A/dm^2^	10–25 at% Sm	magnetic materials	[140]

**Table 7:** Comparison of electrolytes used for electrodeposition of samarium, its alloys and oxide.

Aspect	Aqueous Solutions	Molecular Liquids	Ionic Liquids	Deep Eutectic Solvents	Molten Salts
Solvent Type	Inorganic	Organic	Organic	Organic	Inorganic
Pure Samarium Deposition	No	Yes/No ^1^	Yes	Yes ^1^	No
Samarium Alloy Deposition	Yes ^2^	Yes ^2^	Yes ^2^	Yes ^2^	Yes
Samarium Oxide Deposition	Yes	Possible ^3^	Not reported	Not reported	Not reported
Operation Temperature	20–60 °C	25–60 °C	25–120 °C	70–80 °C	450–750 °C
Deposition Potential (Ag/Ag^+^)	−0.8 to −2.0 V	−2.8 V	−0.5 to −3.5 V	−0.7 to −1.9 V	−1.6 to −2.4 V
Current Density	0.5 to 70 A/dm^2^	1 A/dm^2^	Not reported	0.1 to 0.5 A/dm^2^	5 to 200 A/dm^2^
Advantages	easy to handle, low cost, high conductivity	wider electrochemical windows, pure samarium deposition			high efficiency, thick deposits
Disadvantages	low efficiency, only some alloys ^2^	moisture-sensitive, lower conductivity, higher costs, unknown efficiency			energy-intensive, only for alloys
		volatile, toxic	expensive, toxic	low toxicity	

^1^ Dependent on bath composition and substrate material. ^2^ Known only with iron-group metals. ^3^ Molecular plating of nuclear targets from a mixture of organic solvents (alcohols or acetone) and some water, followed by calcination [159,160].

## Data Availability

No new data were created or analyzed in this study. Data sharing is not applicable to this article.

## Abbreviations

The following abbreviations are used in this manuscript:

SHE	Standard hydrogen electrode
SCE	Saturated calomel electrode
Fc/Fc^+^	Ferrocene/ferrocenium redox couple
XPS	X-ray photoelectron spectroscopy
XRD	X-ray diffractometry
GD	Galvanostatic electrodeposition
PD	Potentiostatic electrodeposition
PCD	Pulsed current electrodeposition
DCV	Direct current deposition at constant voltage
OCP	Open circuit potential
GC	Glassy carbon
